# Prevent infection linked to the dialysis water in a hemodialysis center in Fez city (Morocco)

**DOI:** 10.11604/pamj.2013.16.122.2877

**Published:** 2013-11-28

**Authors:** Bouchra Oumokhtar, Abdelhakim El Ouali Lalami, Mustapha Mahmoud, Sanae Berrada, Mohammed Arrayhani, Tarik Squalli Houssaini

**Affiliations:** 1 Laboratoire de Microbiologie, Faculté de médecine et de pharmacie, Université Sidi Mohammed Ben Abdallah, Fès, Maroc; 2 Laboratoire de Diagnostic épidémiologique et d'hygiène du milieu. Direction régionale de la santé, Fès, Maroc; 3 Service de néphrologie, CHU Hassan II, Faculté de médecine et de pharmacie, Université Sidi Mohammed Ben Abdallah, Fès, Maroc

**Keywords:** Hemodialysis, Water, Dialysate, microbial contamination, disinfection, antibiotic resistance

## Abstract

**Background:**

Water treatment systems are a critical variable in dialysis therapy. Rigorous control of hemodialysis water quality is particularly important in order to guarantee a better quality of life of the hemodialysis patients. The objective of the study was to evaluate the chemical, microbiological quality and antimicrobial resistance of bacteria isolated from water and dialysate in a public HD center.

**Methods:**

Fifty five samples of water and dialysate were collected weekly over a period of 4 months. The samples were collected from 4 points in the distribution loop. The microbiological and chemical analyses were performed according to our national standards. Antimicrobial susceptibilities patterns of isolated bacteria were determined by disk diffusion method.

**Results:**

The chemical and microbiological parameters in all dialysis water and dialysate samples are in accordance with national standards. However, 70 Gram-negative bacteria were identified: *Pseudomonas sp, Ochrobactrum antropi* and *Burkholderia cepacia*, isolated at 52.8%, 12.8% and 17% simultaneously. Fourteen per cent of the isolates were resistant to three or more antibiotics. All resistant bacteria belong to the genus of *Pseudomonas*, 80% were resistant to tetracycline and to co-trimoxazole, 30% to ceftazidime. No colistin and imipenem resistance was observed.

**Conclusion:**

To avoid a health risk due to bacterial contamination, an adequate system for water treatment, disinfection of the hemodialysis system and microbiological monitoring of the water and dialysate are necessary.

## Introduction

Each year, around 4,000 new cases of patients with? End Stage Renal Diseases? (ESRD) treated by maintenance hemodialysis HD are seen in Morocco. In dialysis therapy, HD patients are exposed to 360-600 liters per week of dialysate. Therefore, all the low molecular weight substances present in the water have direct access, through the semi-permeable membrane of the dialyzer, to the dialysis patient′s blood stream [1]. A variety of Gram-negative bacteria can multiply and forming a biofilm in all types of waters and on the inner surfaces of the hemodialysis equipment [2]. Once it has installed, the biofilm is a constant source of bacterial release that is very difficult to completely remove despite regular attempts of disinfection [1, 3]. A low level of biological contaminants in the dialysate has been associated with a chronic inflammatory state [4]. This causes significant long-term morbidity in HD patients and forms part of the larger syndrome of malnutrition, inflammation and atherosclerosis, the so-called MIA syndrome [4, 5]. The inadequate disinfection of the water distribution systems or pipes inside the dialyzers has been incriminated in several outbreaks of Gram-negative bactereamia and pyrogenia in HD units [6, 7]. So, Disinfection protocol must be preventative and frequent to assure a control quality of dialysis unit [8]. Furthermore, it has been reported that in order to assure the management of dialysis water quality, microbiological monitoring, identity and antibiotic resistance profile of the potentially pathogenic bacteria is needed [9]. The aim of this study was to evaluate the chemical, microbiological quality and antimicrobial resistance patterns of Gram-negative bacteria isolated from water and dialysate in a public HD center of Fez (Morocco).

## Methods

### Research site and general conditions

The study was conducted in a public hemodialysis center affiliated to “Al Ghassani” Hospital in Fez, Morocco. The unit has 21 dialysis machines centered on two nursing stations. Two machines are set aside for patients infected with the hepatitis B virus. The center performs approximately 950 hemodialysis sessions each month for the 80 patients suffering from chronic kidney disease and around 100 sessions of acute hemodialysis. The water treatment is conducted in basement of center. Water treatment system includes tap water pre-treatment with filter system, a softener, an activated carbon filter followed by final purification with double reverse osmosis RO process. Distribution piping is installed in an indirect feed style. Treated water is stored in reservoir holding tank from where it is distributed to dialysis machines. Dialysis facilities water treatment is carried out twice per quarter with a mixture of acetic acid, peracetic acid and hydrogen peroxide.

#### Samples

Fifty five samples of water and dialysate were collected weekly over a period of 4 months. The sampling port should be sterilized with alcohol immediately before sampling. The samples (500 ml) was collecting from 4 points in the distribution loop, raw water (n = 4), after reverse osmosis RO (n = 10), before the start of loop (after water storage tank) (n = 10), after the back loop (n = 10), 21 samples from dialysate effluent. The first samples were made immediately after periodic disinfection performed on February 17, 2010. A second disinfection was performed on 14 April. Moroccan standards for dialysis water recommend that sampling should be performed at the output of the RO and the start of the loop distribution for microbiological and chemical analysis.

### Bacterial analysis

The samples used for microbiological testing were collected aseptically in sterile flasks. To estimate the number of heterotrophic plate count bacteria, the membrane filter technique was employed. A volume of 100 ml of the samples were filtered through membrane filters with pores 0.45 mm in diameter (Sartorius^®^). The membranes were then placed face up on standard plate count agar (PCA, Oxoid^®^) and incubated for 72±3 h at 37°C and 22°C. The maximum level of total flora (heterotrophic bacteria) is defined by Moroccan ministerial decree who recommends a threshold of less than 100 colony forming units (CFU)/mL [10]. For the water supply public; total coliforms (TC), faecal coliforms (FC) and intestinal enterococci (IE), total heterotrophic bacteria at 37°C and 22°C for 24 and 72 h respectively, were determined following the national standards [11]. The media employed are tergitol 7 Agar (Oxoid^®^) for TC and FC, Slanetz & Bartley (Oxoid^®^) for IE. Detection of endotoxin was performed by the Limulus amebocyte lysate test [12]. Endotoxin concentration must be less than 0.25 Endotoxin Unit EU/mL [10]. Conventional microbiological methods were used for identification of bacteria isolated from water treatment system and dialysate: Gram, Oxidase, motility and oxidation-fermentation (OF). Oxidative bacteria were identified by the API 20 NE system (bioMérieux, Marcy-L'Etoile, France).

### Antimicrobial resistance

Antimicrobial susceptibilities were determined by disk diffusion method on Mueller-Hinton agar (BD Microbiology Systems, USA). Eight different classes of antibiotics were selected according to the recommendations of CASFM committee [13]: ticarcillin, imipenem, ceftazidime, cefepime, amikacin, gentamicin, tobramycin, ciprofloxacin, colistin, cotrimoxazol, fosfomycin, rifampicin.

### Chemical analysis

Simultaneously to the microbiological tests, chemical parameters (pH, conductivity, hardness, calcium, magnesium, nitrate, nitrite, ammonium, sulphate and free residual chlorine) were measured according to the national standards for drinking water.

### Statistical Analysis

Statistical analysis was done by SPSS 11.5 statistical software. Results are reported as mean ± standard deviation. The p < 0.05 was deemed as statistically significant.

## Results

The chemical parameters in the raw water samples and in the treated dialysis water are in accordance national standards for drinking water [11] and for dialysis water [10]. Total flora counts of tap water are ranged from 0 and 8 cfu/ml, and no faecal indicators bacteria were found. These results are in accordance with national recommendations [11]. As well, the samples of treatment water and dialysate showed counts of heterotrophic bacteria lower than the limit permitted by national standards for water dialysis: 100 ufc/ml (Table 1). We showed statically significant differences between treated water and dialysate (p= 0.02). Endotoxins were identified in 100% of treated water samples with values below the national limit (<0.25 EU/mL).

**Table 1:** Mean values of total bacteria in treated water and dialysate.

	Total heterotrophic bacteria cfu/ml				*P value*
	Reverse Osmosis	check loop	back loop	dialysate	
**Mean values ± SD**	0.74±1.02	5.23±6.87	10.97±7.55	8.74±5.52	0,02

**Figure 1:**
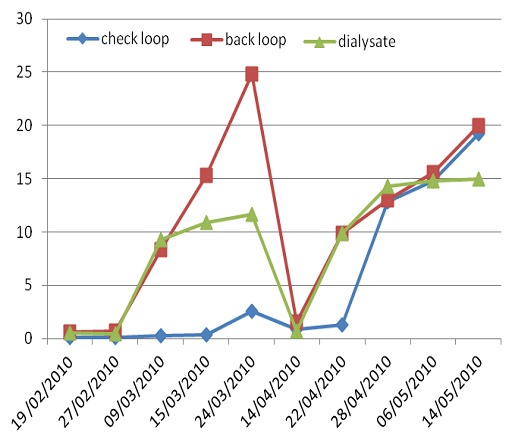
Level of bacterial contamination during the study period showing a rapid increase of bacterial count after the 2sd disinfection of dialysate system

During the study period, monitoring of the level of contamination showed a rapid increase of bacterial count after the 2^sd^ disinfection of dialysate system. A monthly chemical disinfection of the system water has been realized the 14/04/2010 (Figure 1). We noted statically significant differences in contamination level between the first and second period (p= 0.04).

Eighty bacteria were isolated in all water samples and dialysate. Four of them were isolated from tap water, 36 from treated water, and 30 from dialysate samples. Ten isolates have not been able be identified (08 Gram-positive rods and 2 Gram-negative rods). Seventy bacteria were identified as: *Pseudomonas sp, Ochrobactrum antropi*and *Burkholderia cepacia*, isolated at 52.8%, 12.8% and 17% simultaneously. The species of Pseudomonas recovered are: *P. aeruginosa, P. fluorescens, P. stutzeri, P. oryzihabitans* and *P. vesicularis*. Table 2 lists the different bacteria isolated from the samples of water and dialysate. Different antibiotic resistance profiles were registered for bacterial isolates from water and dialysate. Of the 70 Gram-negative isolates examined, 14% were resistant to at least one of the antibiotics examined. All resistant bacteria belong to the genus of *Pseudomonas*, 80% were resistant to tetracycline and to co-trimoxazole, 30% to ceftazidime, 20% to ticarcilline, 16% to amikacin, 12.5% to cefepime and 8% to ciprofloxacin. No colistin, piperacillin and imipenem resistance was observed.

**Table 2:** Bacteria species isolated from water and dialysate samples

Micro-organism	Number of isolates					
	tap water	Reverse Osmosis	Check loop	Back loop	Dialysate	Total
***Burkholderia cepacia***	**1**	**0**	**3**	**4**	**4**	**12**
***Ochrobactrum antropi***	**1**	**0**	**1**	**4**	**3**	**9**
***Acinetobacter haemolyticus***	**0**	**0**	**0**	**2**	**4**	**6**
***Ralstonia pickettii***	**0**	**0**	**1**	**2**	**1**	**4**
***Aeromonas hydrophila***	**1**	**0**	**0**	**1**	**0**	**2**
***Pseudomonas oryzihabitans***	**1**	**0**	**0**	**4**	**5**	**10**
***Pseudomonas vesicularis***	**0**	**0**	**2**	**2**	**4**	**8**
***Pseudomonas stutzeri***	**0**	**1**	**0**	**2**	**5**	**8**
***Pseudomonas fluorescens***	**0**	**1**	**0**	**2**	**3**	**6**
***Pseudomonas aeruginosa***	**0**	**0**	**4**	**0**	**1**	**5**
**Total**	**4**	**2**	**11**	**23**	**30**	**70**

## Discussion

Many patients have to be treated by renal replacement therapies for a long time because renal transplantations are not common in Morocco. We investigated the quality of the mains water and treated water used for dilution of hemodialysis concentrates at a public hemodialysis center in Fez. In this work, all test results physico-chemical, bacteriological and endotoxin were within national guidelines [10]. However among HD patients, clinical studies have confirmed that hemodialysis water, despite acceptable levels of bacterial contamination may cause a pyrogenic reaction and chronic inflammatory state [4, 14]. Microbial contaminants, including fragments of endotoxin, peptidoglycans, and fragments of bacterial deoxyribonucleic acid, can cross both low-flux and high-flux membranes, stimulate cytokine production and trigger elevation of acute phase reaction proteins like C-reactive protein CRP [15]. Several studies have shown that the use of ultrapure water defined as microbial contamination of <0.1 CFU/mL and endotoxin contamination of <0.03 IU/mL, leads to a significant decrease in inflammatory parameters [14, 16]. Using of ultrapure dialysis fluid for all patients and all dialysis modalities was recommended worldwide [9, 17]. In this study, we noted the highest frequency and level of contamination in dialysate. These results suggest that the dialysis machine is the main source of contamination. Also, we noted a rapid increase of bacterial count after the second periodic disinfection of water treatment system. This suggests that despite the disinfection procedure routinely applied, biofilm have been installed in hydraulic circuit of the water treatment and/or of hemodialysis monitors. Nystrand have reported that a microbial analysis result of 5-10 CFU/mL in a system, which is the target for regular disinfection, is already a clear indication that somewhere in the system, microbial growth is in progress [8]. Indeed, it has been demonstrated that the presence of a biofilm on the pipes leads to a rapid regrowth of bacteria after a few hours of disinfection of the water system [18]. In order to remove biofilm, the suggested protocol is chemical disinfection twice a week of reverse osmosis membranes and thermal disinfection daily of distribution piping [8]. In our center, the water treatment system is carried out twice per quarter and no procedures are documented clearly. Furthermore, treated water is stored in reservoir holding tank from where it is distributed to dialysis machines. It was reported that water stagnancy is a contributing factor to bacterial contamination of the water in the pipe systems [19]. More stringent quality control monitoring is necessary to prevent bacterial population in our hemodialysis units. An ultrafiltration membrane placed immediately before the entrance of dialysate into the dialyser has been recommended as a measure against the microbial contamination of dialysate [20].

Several Gram-negative bacilli were isolated both from the water distribution system and the dialysate. We isolated genus *Pseudomonas* at 52.8% in agreement with other authors [21–23]. These bacteria are able to grow rapidly even in sterile water and dialysis fluids. This bacterial growth might be even faster due to the presence of glucose and bicarbonate [23]. *Burkholderia cepacia* isolated at 17% in this study, has been associated with outbreaks of bacteraemia in a hemodialysis units [24]. As previously reported [23], *B. cepacia* was isolated in water samples taken directly from points before and after water treatment and from the kidney machines. Similarly, *Ochrobactrum antropi, Ralstonia pickettii* and *Acinetobacter haemolyticus* were also present in treated water suggesting that the piping system should be a site of biofilm growth, especially on the dialyzers. All *Pseudomonas* isolates in dialysis water were highly resistant to tetracyclines, which have been the most commonly prescribed antibiotics since the 1950s. The same result is reported by other studies [25, 22]. Tetracycline resistance can be mediated by plasmid-encoding genes such as *tet* genes, which encode a tetracycline efflux system or protect ribosomes from the action of tetracycline [26]. These genes may are also sometimes associated with conjugative plasmids, suggesting that the acquisition of these elements with drug resistance genes by *P. aeruginosa* increases difficulty in treating infectious diseases, including nosocomial infections caused by these bacteria. The imipenem which belong to the carbapenems group, was still highly effective. In contrast, we noted that 30% of all *Pseudomonas* sp. was resistant to ceftazidime. This result is disquieting as this is one of the much effective antibiotics against both Gram-negative and Gram-positive bacteria.

## Conclusion

In this study, indeed the results of microbial analysis are below the national standards, we isolated a several bacteria, comprising resistant to antibiotics, from the water used in the dialysis fluid and in the kidney machine. These results suggest the need for continual monitoring of the water supplies in hemodialysis centers and the adoption of effective prophylactic measures that minimize the exposure of these immunodeficient patients to contaminated sources of water.
